# Pectoralis major muscle flap reconstruction after a total laryngectomy widened anteriorly: A case report

**DOI:** 10.1016/j.ijscr.2024.109665

**Published:** 2024-04-20

**Authors:** B. Saout Arrih, Y. Oukessou, W. Bijou, R. Abada, M. Mahtar

**Affiliations:** Department of Otolaryngology, Head and Neck Surgery, Ibn Rochd University Hospital, Faculty of Medicine and Pharmacy, Hassan II University Casablanca, Morocco

**Keywords:** Pectoralis major flap, Laryngeal tumor, Reconstruction, Case report

## Abstract

**Introduction and importance:**

The pectoralis major flap is one of the leading regional pedicled flaps for reconstructive surgery of the head and neck, particularly in oncology after tumor resection. Despite the increasing use of free flaps, this type of flap is still highly recommended in defined indications. It is a highly reliable flap in terms of viability.

**Case presentation:**

We report the case of a man treated in our ENT department for a squamous cell carcinoma of the three laryngeal stages, anteriorly very extensive and requiring total laryngectomy extended to the skin and subcutaneous planes opposite, followed by reconstruction with a flap of the pectoralis major muscle. Post-surgical outcome was excellent.

**Clinical discussion:**

Aryan was the first person to describe the use of the pectoralis major muscle flap. Since then, several studies have demonstrated the great value of this flap in face and neck reconstruction, thanks to its wide indications and excellent viability rate.

**Conclusion:**

The pectoralis major muscle flap represents a valuable reconstructive option for substance loss in the cervicofacial region despite the great development of microsurgery and free flaps.

## Highlights

• The axial vascularization makes the pectoralis major flap a highly reliable flap, with a viability rate of up to 98%.
• This flap's volume is highly advantageous for reconstruction of the neck and lower third of the face after major tumor resection, especially for patients with cancer.
• It is a flap with a simple surgical technique based on the preservation of the nourishing pedicle of the pectoralis major muscle.
• The pectoralis major muscle flap is still recommended in numerous cases, despite the rise of microsurgery and free flaps.

## 1 Introduction

In general, three types of flaps can be differentiated. The local flap, which is a tissue harvested in the immediate vicinity of the defect to be repaired (e.g., advancement and transposition flaps). The pedicled flap, which does not originate from the immediate surroundings of the area to be covered. In fact, the tissue remains attached to its harvesting site by a pedicle that provides it with its blood supply, thus making reference to the “tunnelling” of the flap, which consists of passing the flap through a canal from the harvesting point. The free flap, which is completely excised from the distant donor site, requires reattachment to the recipient site via microvascular anastomoses.

Improved microsurgical techniques have made free flaps more prevalent over the years. Yet failure of a free flap is sometimes unavoidable, especially in patients with underlying vascular conditions. This makes the selection of an appropriate reconstructive method to fill residual defects for these patients a challenging task. Pedicled flaps for such patients, like the pectoralis major myocutaneous flap, are useful options for cervical and maxillo-facial reconstruction [1]. The pectoralis major myocutaneous flap was first introduced by Aryan, and has been used extensively for reconstructive surgery of the head and neck, with a rather long pedicle that includes the thoracoacromial artery as an axial vessel reaching all the cervicofacial regions, and a high-volume flap allowing reconstruction of large loss of tissue, as is often the case when performing surgery on extended tumors of the face and neck [1,2]. In this context, we report the case of a reconstruction involving loss of cervical tissue using the pectoralis major muscle flap after a total laryngectomy extended to the skin and the infrahyoid muscles opposite.

This case report has been reported in line with the SCARE criteria [10].

## 2 Case report

This is a study of a 53-year-old male patient operated on for a laryngeal tumor in our ENT and cervico-facial surgery department. The patient was referred to us from another hospital. It should be noted that the patient was a chronic smoker and suffered from ischemic heart disease and high blood pressure.

**Fig. 1:**
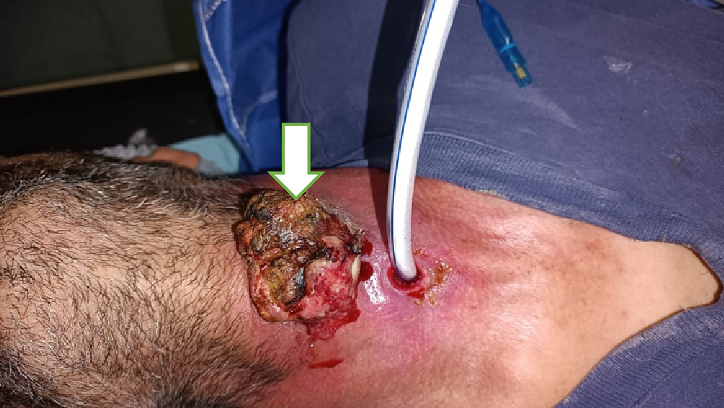
Image showing externalization of the laryngeal tumor.

The history of the disease dates back 2 years to the onset of chronic dysphonia complicated 1 year later by inspiratory dyspnea requiring emergency tracheotomy (Fig. 1). On physical examination, a median cervical ulcerating mass was noted, without other associated signs, notably the patient had no dysphagia and no lymphadenopathy (Fig. 1).

**Fig. 2:**
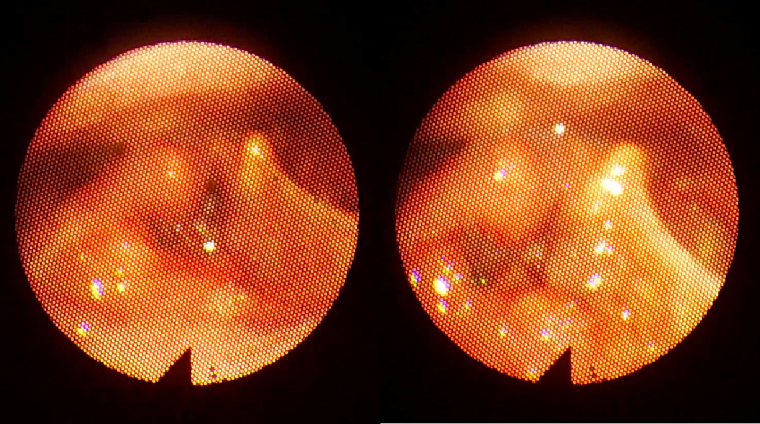
Image showing the endoscopic appearance of the laryngeal tumor.

Nasofibroscopy reveals a mass that obstructs totally the glottic airway, occupying the glottic and supraglottic stages of the larynx, with both vocal cords fixed (Fig. 2).

**Fig. 3:**
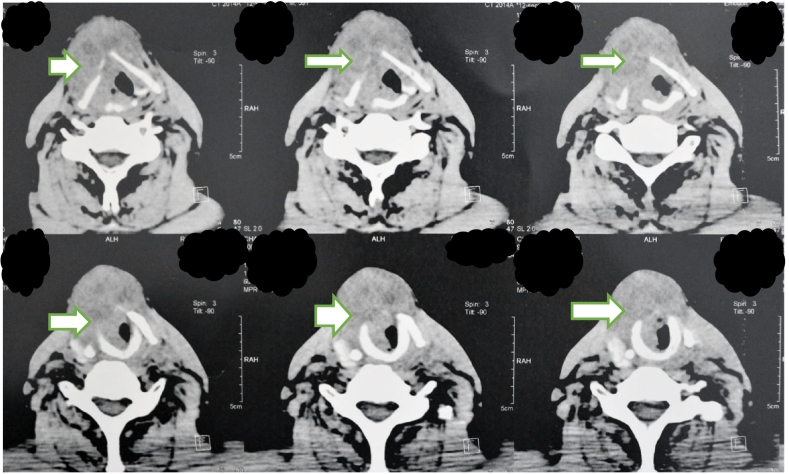
Cervical CT scan showing a laryngeal tumor occupying all three laryngeal stages.

The cervical CT scan revealed a voluminous laryngeal process occupying all laryngeal subunits (supraglottic, glottic, subglottic), measuring 7 cm in long axis and invading the anterior commissure, lysing the thyroid, cricoid and arytenoid cartilages (Fig. 3).

After being stabilized, the patient underwent suspension laryngoscopy with tumor biopsy. This revealed a tumor of all three laryngeal stages, extending far forward, beyond the anterior commissure, invading the infrahyoid muscles, the subcutaneous layers and externalizing through the skin. The tumor did not extend into the piriform fossa or the post-cricoid region, and the entrance to the esophagus was unobstructed. The base of the tongue was not invaded.

Pathological examination is consistent with undifferentiated squamous cell carcinoma.

**Fig. 4:**
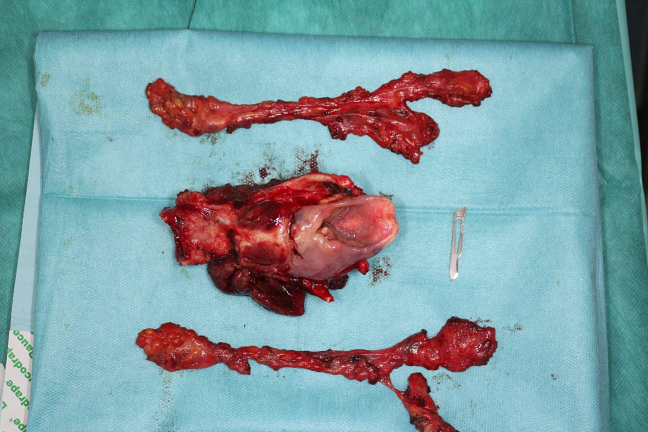
Image showing the total laryngectomy specimen.

**Fig. 5:**
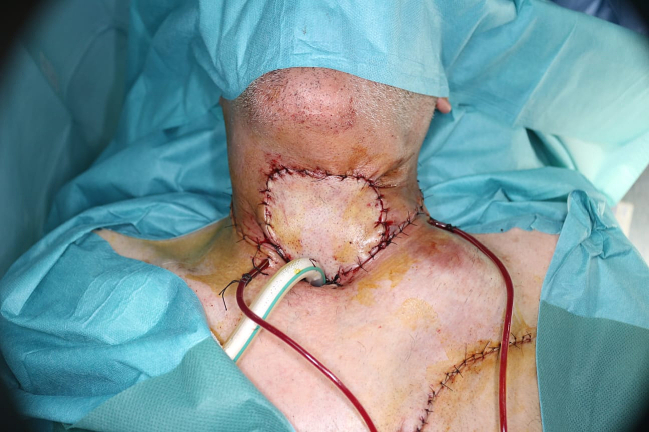
Image showing reconstruction of cervical tissue loss using a pectoralis major muscle flap.

The decision adopted at a multidisciplinary consultation meeting was to perform a total laryngectomy extended anteriorly to the infrahyoid muscles and the skin opposite, with bilateral cervical lymph node dissection and reconstruction of the tissue defect using a flap of the pectoralis major muscle, followed by adjuvant radiotherapy. The choice of this type of flap is based on the patient's cardiovascular condition and the large volume of the tumor, which may compromise the viability of a free flap (Fig. 4, Fig. 5).

**Fig. 6:**
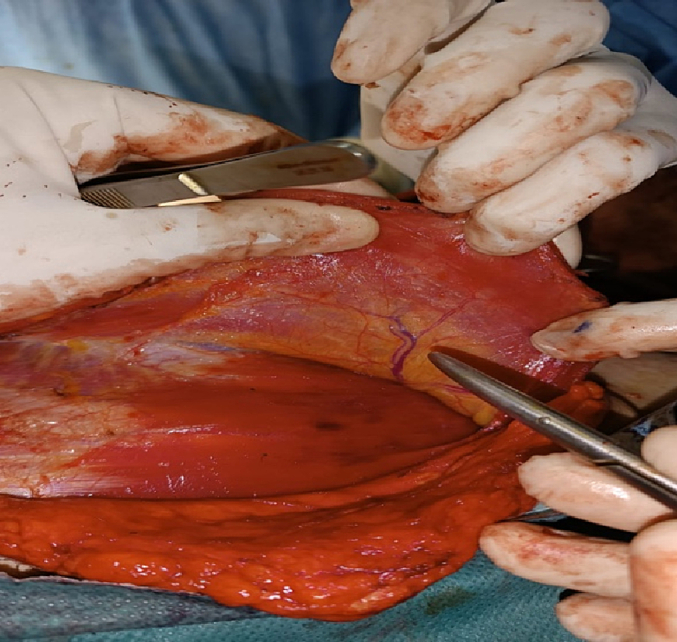
Image showing the nourishing pedicle of the pectoralis major muscle.

The surgery was performed by an ENT specialist, performing a wide dissection of the peripheral skin and subcutaneous tissue over the skin paddle of the pectoralis major muscle up to the clavicle to expose the pectoralis major muscle and facilitate skin suturing, once the flap is made. The main objective when raising the flap was to clearly visualize the nourishing pedicle located on the deep surface of the pectoralis major muscle, and to preserve it during dissection and translation of the flap towards the cervical region (Fig. 6).

On definitive anatomopathological examination, we identified a squamous cell carcinoma of the larynx, invading the thyroid cartilage and infrahyoid muscles. Bilateral lymph node metastases were present, the largest measuring 2 cm on the right and 3 cm on the left. In the light of these data and the preoperative evaluation, we can classify this tumor as T4aN2cM0.

In the immediate postoperative period, we prescribed antibiotics, analgesics and cough suppressants to avoid infection and pressure on the flap's blood circulation. On the second postoperative day, the suction drains were removed. The patient stayed in our department for 7 days after surgery, with daily follow-up, where we checked the color and warmth of the flap to detect any incipient necrosis requiring our emergency intervention, and changed the dressing once a day, making sure it wasn't compressive.

Once the patient has left our department, the patient was advised to stop smoking, and to adopt regular physical activity along with a healthy diet, the aim is to achieve a satisfying blood circulation. We've established a follow-up plan involving consultations every 15 days for the first two months, then every month for the rest of the year. The sutures were removed by the doctor on the 15th postoperative day. All check-ups were favorable.

## 3 Discussion

The pectoralis major flap, as its name suggests, includes the pectoralis major muscle, with or without the skin opposite, and may even include the underlying ribs. It is one of the best-known regional pedicled flaps for reconstructive surgery of the head and neck, especially in oncology after tumor resection in the same operation [1]. It's a highly reliable flap, with a viability rate of up to 98 %, thanks to its axial vascularization based superiorly on the pectoral branch of the thoracoacromial artery. The principle is that a paddle of the pectoralis major muscle, with or without the skin opposite, is lifted and translocated to the cervico-facial region [2]. The pectoralis major muscle flap is a pedicled flap. Three main types can be differentiated. The first is the muscle flap completely covered by the skin paddle. The second is the island muscle flap, which requires dissection of the muscle upstream of the skin paddle, which may result in injury to the main pedicle, but does allow good mobility. The third is the type most commonly used today, with a long muscle paddle covered distally by a skin paddle, it allows reconstructing large losses of muscular and mucosal substance without an unusable volume of skin, this is the type used in the reported case [3,4]. Historically, Hueston in 1968 was the first to describe a technique combining pectoralis major muscle and skin, calling it a composite pectoral flap. But the basis of the pectoralis major muscle flap as we know it today was established by Aryan in 1979, whose technique used the skin of the anterior thorax overlying the pectoralis major muscle with its underlying axial vascular pedicle [5]. It is the flap to choose for patients with cancer requiring adjuvant radiotherapy. The volume of this flap is highly advantageous for musculocutaneous reconstruction of the neck and lower 1/3 of the face after major tumor resection, particularly of the buccal floor. Interestingly, the muscular surface becomes mucosialized even without the use of a skin paddle [6]. It is also indicated in patients with underlying cardiovascular conditions in whom the failure rate of free flaps is very high, as well as in salvage reconstruction after free flap failure. The only absolute contraindication is radical axillary lymph node dissection. Other contraindications that may limit the use of this flap include a history of breast surgery, which may reduce the quality and quantity of musculocutaneous perforating arteries. Poland syndrome, defined by the unilateral absence of the pectoralis major muscle. Morbid obesity with excessive adipose tissue that can compromise the survival of the skin palette. Also, smoking, diabetes and poor nutritional status are suspected of reducing flap survival. The major disadvantage of this flap is its unaesthetic appearance, especially for thin patients [7]. In addition to the advantage of being rapidly preformed, they are viable and reliable, and located close to the resection site in cases of reconstruction after enlarged total laryngectomy. They enable reconstruction in a single procedure, with low complication rates at the donor site. This avoids the need for salvage surgery if performed as a first-line procedure. However, it can also be used as a salvage procedure if the free flap fails [13]. This myocutaneous flap can reduce salivary fistula incidence rates from 24 % to 0 % according to Patel and Keni, and from 51 % to 20 % according to Righini. This goes well with the case reported, which has evolved excellently: the flap has not necrotized and no complications have occurred, especially no infection or salivary fistula despite advanced TNM tumor classification [11,12]. A study evaluated 220 patients and identified several risk factors directly linked to most of the complications that can occur. Hematomas are correlated with advanced tumor stage and, consequently, more radical surgery. Infections were more prevalent in patients with hemoglobin <10 g/dL, serum albumin <3 g/dL, and the presence of underlying systemic disease. Infections also increased significantly over the duration of hospitalization. The risk of fistula increased with more extensive resection, and with a bulky flap. Flap necrosis appears to be more frequent in women than in men, and in patients with underlying conditions [8]. Indeed, according to several studies, the rate of total flap necrosis remains very low, as in a study which reports a rate of only 2 % in a cohort of 500 cases [9].

## 4 Conclusion

In conclusion, we emphasize that the pectoralis major muscle flap still retains its indications despite the development of microsurgery and free flaps. This is mainly due to its high viability rate, the large musculocutaneous volume it offers for reconstructing large losses of substance, its rich vascular supply, its wide rotation arc and also its proximity to different areas of the face, oral cavity and cervical regions.

## Provenance and peer review

Not commissioned, externally peer-reviewed.

## Consent

Written informed consent was obtained from the patient for publication of this case report and accompanying images. A copy of the written consent is available for review by the Editor-in-Chief of this journal on request.

## Ethical approval

Ethical approval was provided by the authors institution.

## Funding

None.

## Guarantor

Saout Arrih Badr.

## Research registration number

N/a.

## CRediT authorship contribution statement

Saout Arrih Badr: Study concept and writing the paper
Oukessou Youssef: Study concept and correction of the paper
Bijou Walid: Study concept and correction of the paper
Rouadi Sami: Study concept and correction of the paper
Abada Reda: Study concept and correction of the paper
Mahtar Mohamed: Study concept and correction of the paper.

## Declaration of competing interest

None.
